# Better Late Than Never: The Impact of Steroidal Treatment on the Outcome of Melanoma Patients Treated with Immunotherapy

**DOI:** 10.3390/cancers15113041

**Published:** 2023-06-03

**Authors:** Neta Bar-Hai, Guy Ben-Betzalel, Ronen Stoff, Shirly Grynberg, Jacob Schachter, Ronnie Shapira-Frommer, Nethanel Asher

**Affiliations:** 1 Ella Lemelbaum Institute for Immuno-Oncology, Sheba Medical Center, Ramat Gan 52621, Israel; 2 Sackler Faculty of Medicine, Tel Aviv University, Tel Aviv 6997801, Israel

**Keywords:** immunotherapy, corticosteroids, melanoma, immune-related adverse events, priming-phase

## Abstract

**Simple Summary:**

In the era of immunotherapy, advanced melanoma patients have unprecedentedly higher survival rates. However, the application of immunotherapy may lead to immune-related adverse events (irAEs), which are often managed with steroids. The potential negative impact of steroids on the effectiveness of immunotherapy remains uncertain, as studies often report contradictory results. This retrospective analysis of 415 first-line immunotherapy patients showed that steroid exposure increased progression-free survival (PFS) compared to no exposure, but early initiation (within four weeks) was linked to a shorter PFS compared to late or no exposure. This suggests that administering steroids during the immunotherapy priming phase may hinder the establishment of an effective immunotherapy-induced immune response. The implications of these findings are significant for managing irAEs, indicating a need for the cautious use of steroids in the initial stages of treatment.

**Abstract:**

Background: Successful treatment with Immune Checkpoint Inhibitors (ICI) requires the balanced activation of the immune system. Over-activation may result in immune-related adverse events (irAEs), which often require steroidal treatment. This study examined the possible impact of steroids on treatment efficacy in melanoma patients concerning initiation timing and dosage. Methods: A retrospective, single-center analysis of patients with advanced melanoma who underwent first-line ICI therapy during 2014–2020 was conducted. Results: Among the 415 patients, two-hundred patients (48.3%) were exposed to steroids during the first line, most of them due to irAEs (*n* = 169, 84.5%). Nearly a quarter of them were exposed to steroids within the first four weeks of treatment. Surprisingly, steroidal exposure was associated with better progression-free survival (PFS; HR = 0.74, *p* = 0.015); however, early exposure (within four weeks of treatment) resulted in a significantly shorter PFS compared to late exposure (adjusted HR 3.2, *p* < 0.001). Conclusions: Early exposure to corticosteroids during the priming phase of ICI therapy could impede the establishment of an effective immune response. These results suggest that caution should be exercised when considering the use of steroids for the management of early-onset irAEs.

## 1. Introduction

The advent of Immune Check-point Inhibitors (ICIs) has led to a significant improvement in the prognosis of patients with advanced or metastatic melanoma, transforming the disease from a poorly responsive condition to one with an impressive response rate. This has resulted in a marked increase in survival rates and an improvement in the quality of life of patients [1,2,3,4,5]. The inhibition of the programmed cell death protein 1 (PD-1) and cytotoxic T-lymphocyte-associated protein 4 (CTLA-4), as key immune checkpoint inhibitors, have been associated with a 6.5-year survival rate of 48% in patients with metastatic melanoma. This survival curve has a typical plateauing which begins at 2 y, and the median duration of response has not yet been reached at the 6.5 y landmark [6].

The expanding use of ICIs in clinical practice, however, is associated with a substantial challenge, secondary to the over-activation of the immune system, causing immune-related adverse events (irAEs) [7,8]. The Criteria for Adverse Events (CTCAE) Grade 3 and 4 irAEs were experienced by 54% of patients who received the combination therapy of ipilimumab and nivolumab (anti-CTLA-4 and anti-PD-1, respectively) in CheckMate 067 [9], and a fatality rate of 1.23% was estimated in the meta-analysis from 2018 [10].

Many studies have investigated the potential association between irAE- associated factors and therapeutic outcomes. Specifically, there was an evolving body of evidence linking the development of irAEs with better survival, suggesting a correlative relation between autoimmunity and the anti-tumor effect elicited by ICIs [11,12,13,14]. Other studies question whether the affected organ, the severity of irAE, the timing of onset and the type and duration of therapeutic intervention could all have a theoretical role in patients’ survival [11,15,16]. Interestingly, of the various irAEs that were found to be associated with enhanced survival, dermatological irAEs, particularly rash and vitiligo, showed the most dependable data [17,18,19,20,21,22]. In addition, thyroid dysfunctions induced by anti-PD-1 seemed to be associated with a better response and increased overall survival (OS) [21,22,23]. It was also found that patients experiencing multiple irAEs, compared to those with a single irAE, had longer OS rates [24]. As for treatment discontinuation due to irAEs, in a recent update on Checkmate 067, 77% of the patients treated with combinatorial immunotherapy that was alive at data cutoff were also treatment free (off treatment, without having received any subsequent systemic therapy). Most of them discontinued their treatment due to irAE. Among the patients who discontinued treatment, the median treatment-free interval was 27.6 months. Many retrospective studies have also shown encouraging efficacy among patients who discontinued immunotherapy due to irAE at any time [25,26,27,28,29], even during the induction phase [14].

The management of irAEs is based mainly on corticosteroids due to their strong and rapid anti-inflammatory properties. Corticosteroids possess potent immunomodulatory activities, including the ability to negatively regulate the activation, differentiation, proliferation and migration of naive T cells [30,31]. They inhibit the production of inflammatory mediators and repress leukocyte recruitment to the tissues. Corticosteroids also increase the activity of regulatory T-cells and thus promote the resolution phase of inflammation. National and international guidelines recommend corticosteroid usage for the routine management of irAE, including the National Comprehensive Cancer Network [32], the European Society of Medical Oncology [33], and the Society for Immunotherapy of Cancer (SITC) Toxicity Management Working Group [34].

The impact, however, of immunomodulatory agents on the therapeutic outcome and survival of patients undergoing ICI treatments remains largely obscure. It should be noted that patients receiving more than 10 mg of prednisolone, which is equivalent at baseline, and those experiencing severe irAEs requiring a high-dose or prolonged steroidal therapy are typically excluded from clinical trials.

Whether the use of steroids and other immune suppressants could hamper the efficacy of cancer immunotherapy has been evaluated in several studies [35,36,37,38,39,40,41,42,43]. Patients with different types of malignancies (mostly lung cancer, renal cell carcinoma and melanoma) when treated with ICI were evaluated in observational multi-center retrospective studies and in a meta-analysis on published trials, all treated with corticosteroids—some at the baseline prior to ICI treatment initiation and some during treatment for various indications. Most studies showed reassuring results with no correlation between the systemic corticosteroids and outcome, concluding that steroid use was not associated with a loss of efficacy in ICIs [36,37,43]. Other studies, however, did find a detrimental effect of steroids on ICI efficacy [38,39,40,41,42]. Specifically, baseline steroids, when administered within the 30 days prior to immunotherapy initiation for cancer-related indications, were the only baseline medication concordantly related to a poorer response rate, PFS and OS in 1012 patients [39]. Additional studies confirmed that the baseline corticosteroids used were indeed associated with poorer disease control [40,42], yet the main negative effect on OS was found in cases where the indication of steroids included symptoms such as palliations, radiation therapy or central nervous system metastases, whereas steroids used to mitigate adverse events did not negatively affect OS [40]. These findings raised the question of whether the negative effects observed with the use of baseline steroids for palliative care and well-being were the result of the patient’s frailty or of the immunosuppressive properties of steroids, which lowered the immune anti-tumoral effect.

In this study, we investigated the effects of corticosteroid exposure during the first-line immunotherapy treatment in patients with advanced melanoma at our institute. We explored which factors were related, if any, to the outcome.

## 2. Materials and Methods

### 2.1. Patients and Study Design

This is a real-world single-center retrospective study based on the institute’s melanoma database registry. The study cohort comprised unresectable or metastatic AJCC Stage IV cutaneous melanoma who were treated with immunotherapy in the first-line setting at the Ella Lemelbaum Institute for Immuno-oncology, Sheba medical center, Israel, between 2014 and 2020. Study data were collected and managed using REDCap electronic data capture tools hosted at the Sheba medical center: a secure software platform to support data capture and export procedures [44]. Demographic details, the clinical features of baseline characteristics, data regarding first-line treatments and radiologic responses were collected from source medical files according to the oncologists’ definitions. irAEs were graded according to the Common CTCAE v.5.0. Data regarding steroidal treatments were meticulously collected and included: doses expressed in mg/kg of the prednisolone equivalent, routes of administration, timing, duration, and exposure. Exposure to steroids was calculated as the doses per week of exposure and was divided by two due to a theoretical linear tapering down. Disease progression and survival data were used to estimate the median PFS and OS.

### 2.2. Statistical Analysis

OS and PFS were estimated from the initiation of immunotherapy to death and to progression or death, respectively. We used Kaplan–Meier methods to estimate and visualize survival and Cox proportional hazards regressions to assess an association with baseline prognostic factors. For the time-dependent variable “onset of steroidal initiation”, a landmark analysis was also carried out to explore the presence of any bias related to this variable. All clinical variables that resulted significantly in univariable analysis for PFS were tested in multivariable analysis. Statistical significance was defined as a *p* ≤ 0.05 level, and all tests were two-sided. All analyses were performed with STATA v.13.0.

### 2.3. Ethics

This single-center, retrospective study of medical records was approved by the Institutional Review Board of the Sheba Medical Center (4387-17-SMC).

## 3. Results

Four hundred and fifteen (415) patients diagnosed with advanced melanoma were treated at Sheba medical center with immunotherapy between 2014 and 2020. The baseline demographic data and treatment dispositions are described in Table 1.

The median age was 68 years (range 12–99), and 58% (*n* = 241) were males. Treatments in the first line were anti-PD-1 monotherapy (*n* = 271, 65.3%)—either Nivolumab or Pembrolizumab—a combination of anti-PD-1 and anti-CTLA-4 (*n* = 101, 24.3%) and anti-CTLA-4 monotherapy (*n* = 43, 10.4%). Most of the cohort had cutaneous melanoma (*n* = 326, 78.5%). A minority of 5.8% (24 patients) had a baseline auto-immune disease, in which five patients (1.2%) were treated with chronic immune suppression (prednisone -3, mesalamine -1, azathioprine-1). Eastern Cooperative Oncology Group Performance Status (ECOG PS) at first-line treatment initiation was 0 in 274 patients (68.5%) and 1 in 85 patients (21.2%). Accordingly, the LDH level was elevated in 20.5% of the patients (*n* = 85). The BRAF mutational status was wild type in 62.4% (*n* = 259) and mutant in 29.4% (*n* = 122).

After a median follow-up of 24.5 months (IQR 9.2–41 months), the median treatment duration was 6 months (range 0.1–61 months). The reasons for treatment discontinuation were disease progression (*n* = 193, 46.5%), toxicity (*n* = 86, 20.7%), and durable deep response (*n* = 50, 12.1%). In 44 patients (10.6%), treatment was ongoing. The response rates to anti-PD-1 monotherapy, with a combination of anti-PD-1 and anti-CTLA-4 and anti-CTLA-4 monotherapy, were 58.3%, 59.4% and 20.5%, respectively.

The median progression-free survival for patients treated with anti-PD-1 monotherapy, including a combination of anti-PD-1 and anti-CTLA-4 and anti-CTLA-4 monotherapy, were 12.5 months, 12.8 months and 3.4 months, respectively. The median overall survival for patients treated with anti-PD-1 monotherapy with a combination of anti-PD-1 and anti-CTLA-4 and anti-CTLA-4 monotherapy was 41.3 months, not-reached and 55 months, respectively.

### 3.1. Immune-Related Adverse Events (irAEs)

During the treatment and follow-up periods, the irAEs of any grade were seen in 299 patients (72%) and 96 patients (23%) who experienced no AEs. Data regarding irAE were missing in 20 patients (5%). High-grade AEs 3–5 were seen in 104 patients (25%), and low grade AEs 1–2 were seen in 142 patients (33%). For 73 patients (18%), the grade of the AE was not documented. There was only one case of fatal (Grade 5) pneumonitis, secondary to treatment with ipilimumab and nivolumab.

**Figure 1:**
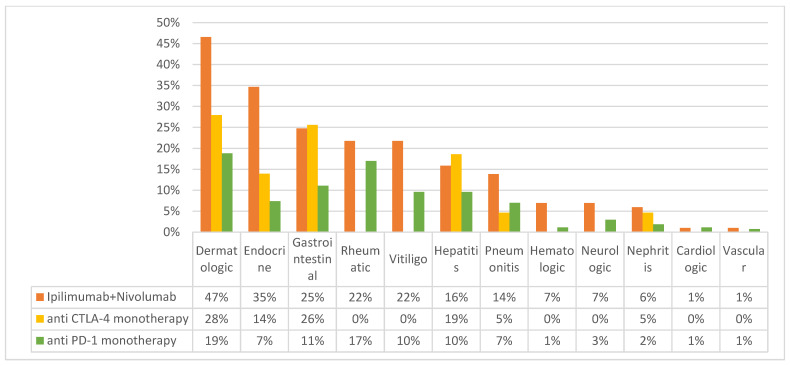
Frequency of immune related adverse events by protocol. Comparison of immune-related adverse event (irAE) rates across different immunotherapy protocols, classified by the type of irAE. Anti PD-1 monotherapy—either Nivolumab or Pembrolizumab.

High-grade irAEs (grade 3–5) were experienced by 60.9% of patients treated with a combination of anti-PD-1 and anti-CTLA-4, 30.5% of patients treated with anti-CTLA-4 alone, and 16.8% of patients treated with anti-PD-1. See Figure 1 for more details.

### 3.2. Steroid Exposure

In total, 200 patients (48.3%) were exposed to steroids during the first line, most of them due to irAEs (*n* = 169, 84.5%). Other reasons were Central Nervous System (CNS) metastasis (*n* = 24, 12%), well-being (*n* = 5, 2.5%) and chronic low-dose medication (*n* = 2, 1%).

An impressive fraction of the patients treated with the ipilimumab and nivolumab combination were exposed to steroids during their course of treatment (77%, *n* = 77), while 39.5% (*n* = 107) and 37% (*n* = 16) of the patients treated with anti-PD-1 monotherapy (either Nivolumab or Pembrolizumab) and anti-CTLA-4 monotherapy, respectively, were exposed to steroids.

The route of administration was oral in 157 patients (78.5%) and intravenous in 34 (17%). The median **dose** of the steroids was 0.75 mg/kg of a prednisolone equivalent (range 0.03- 80 mg/kg), with a median **duration** of steroidal treatment for 11.5 weeks (range 0.1–316 w). The median **exposure** was 4.11 mg/kg/week (range 0.1–5074).

**Figure 2:**
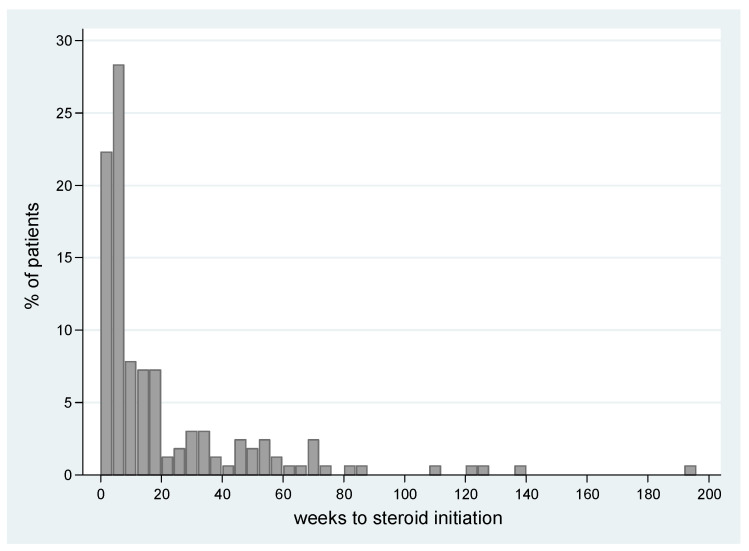
Onset of steroidal exposure relative to immunotherapy commencement. The proportion of patients who received steroids were categorized by the timing of their steroid treatment in relation to the commencement of immunotherapy.

The median time to **onset** of the steroidal treatment for irAEs was at 7.8 weeks from immunotherapy initiation (range 0–193 w), with almost a quarter of the patients (23%, *n* = 38) initiating steroidal treatment within the first 4 weeks of treatment (for a histogram distribution see Figure 2).

Additional immune-suppressive treatments were administered to 16 patients (8%). These were infliximab (8 patients), IV immunoglobulins (4), mycophenolate mofetil (2), sulfasalazine (2), budesonide (1), plasma exchange (1), cyclosporine (1) and mesalamine (1).

### 3.3. irAEs, Steroid Exposure and Treatment Efficacy

Compared to patients with no documented irAE, patients who experienced irAE, irrespective of its severity grade, had a significant PFS benefit with HR 0.46, *p* < 0.001 (95%CI 0.35–0.6). In accordance with this finding, patients who were exposed to steroids during the first line of treatment also had a significant PFS benefit over those who were not exposed to steroids, with a 26% reduction in the risk of disease progression or death [HR 0.74, *p* = 0.015 (95%CI 0.58–0.94)].

Looking into the characteristics of steroid administration, we found no association between the dose of steroids and PFS (HR 0.98, *p* = 0.32). Due to the intrinsic bias associated with time-dependent events, we were not able to estimate the effect of the duration of steroidal treatment on PFS, nor the effect of exposure (defined by dose per duration divided by two) on PFS.

### 3.4. Timing of Steroid Exposure

**Figure 3:**
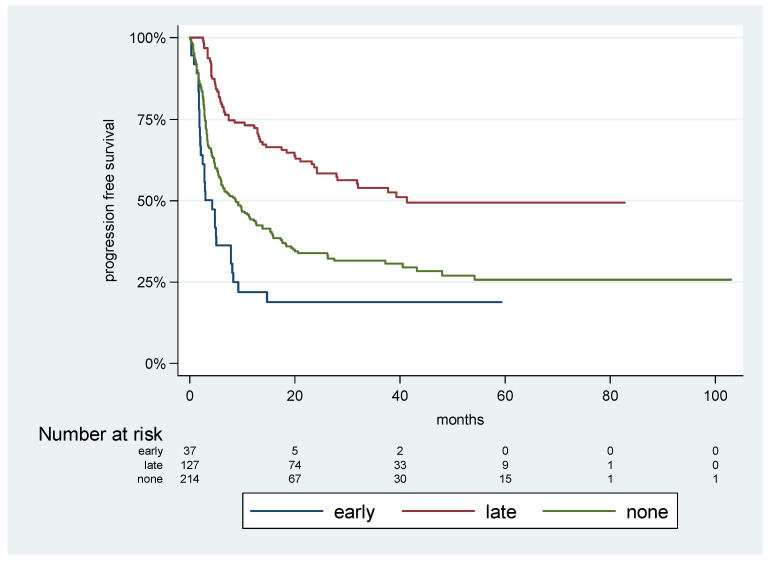
Survival analysis according to timing of steroidal exposure Progression-free survival with Kaplan–Meier analysis according to timing of steroid exposure: “Early”—patients who were exposed to steroids within the first 4 weeks of immunotherapy. “Late”—patients who were exposed to steroids after more than 4 weeks from immunotherapy initiation. “None”—Patient who were not exposed to steroids. The median PFS were 4.2 m for “Early” cases, 8.75 m for “None”, and 41.3 m for “Late”.

Looking at the onset of steroid treatment in relation to the immunotherapy initiation, we found that patients who were exposed to steroids in the first 4 weeks of immunotherapy treatment (“early”) had a significantly shorter PFS compared to those who were exposed to steroids later during the course of treatment (“late”), and even compared to patients who were not exposed to steroids at all (“none”). The median PFSs were 4.2 m for “early” cases, 8.75 m for “none”, and 41.3 m for “late”. See Figure 3 for the PFS curves.

In order to avoid a possible “immortal time bias”, which was attributed to a longer follow-up in patients who were exposed to steroids later in the course of the disease, we analyzed the effect on PFS, adjusting for a 6-month landmark. The adjusted HR (adjHR) resulted in a significant outcome for “early” cases compared to “late” cases, the adjHR was 3.2 (*p* < 0.001, 95%CI 2.0–4.9), and for the “none” cases compared to the “late” cases, the adjHR was 2.0 (*p* < 0.001, 95%CI 1.5–2.8).

### 3.5. Univariable and Multivariable Analysis

On the univariable analysis for PFS, the clinical factors which resulted in being statistically significant were cutaneous histology, the M sub-stage, ECOG-PS, relative LDH level, anti-PD-1 containing treatments (vs. anti-CTLA-4 alone), and development with irAE and the onset of steroidal treatment initiation. On the multivariable analysis for PFS, the onset of steroidal treatment remained significant at HR 2.45 for early onset compared to late-onset (95%CI 1.26–4.74, *p* = 0.008). Other significant prognostic variables included a higher M sub-stage [HR 1.28 (95%CI 1.04–1.57, *p* = 0.02)], relative LDH level [HR 1.19, (95%CI 1.08–1.32, *p* = 0.001)], immunotherapy protocol [HR 0.68 for anti-PD-1 containing treatments vs. anti-CTLA-4 alone, (95%CI 0.46–0.99, *p* = 0.049)] and the development of irAE (HR 0.48 (95%CI 0.31–0.75, *p* = 0.001)]. See Table 2 for uni- and multivariable analysis details.

## 4. Discussion

Allowing the immune system to re-activate and regain dominance over tumor progression, immunotherapy has changed the prognosis of countless patients in an unprecedented way. This unique mechanism of action can, however, result in a loss of self-tolerance and the subsequent development of irAEs. The incidence and the severity of irAE varies, depending on the type of immunotherapy used, and can vary from mild skin rashes to life-threatening conditions such as colitis and hepatitis. irAEs are often managed with corticosteroids, which possess strong and rapid anti-inflammatory activities. Certain irAEs require prolonged immune suppression, which is particularly rheumatologic, dermatologic, and occasionally hepatic and respiratory, and can be potentially steroid-resistant.

Some studies have suggested that the immune suppressive properties of corticosteroids might diminish the immune system’s anti-tumor effect and contribute to the emergence of primary or secondary resistance to immunotherapy. Therefore, the clinical application of corticosteroids has become a subject of discussion, with questions arising regarding the appropriate dosage and timing of treatment. In this context, we aimed to address the uncertainty surrounding the use of corticosteroids for the management of irAEs induced by ICIs.

In this study, we analyzed data from 415 patients with advanced melanoma who received immunotherapy in the first line and were followed up for a median of two years. As expected, 72% of the patients experienced irAEs of any grade, with 25% of these experiencing CTCAE grades 3–5 irAEs. Regardless of irAE severity, our findings revealed that patients who experienced an irAE had a significantly better outcome compared to patients who did not experience any irAE. Essentially, experiencing irAE was associated with a lower probability of disease progression or death (HR 0.46). These results were aligned with previous studies [11,12,13,45,46,47,48,49,50,51,52,53,54] and supported the theory that an intrinsic development of an irAE may represent an important marker of immune activation and response to treatment.

Looking at the progression or death probability among patients who were exposed to steroids during the first line (48.3% of the study cohort) compared to those who were not exposed, we found no association between exposure to steroids and a compromised PFS. In fact, patients who were exposed to steroids demonstrated a longer PFS with a 26% reduction in the risk of disease progression or death (HR 0.74). Furthermore, our data showed no negative impact on PFS in relation to the dose of steroids used.

Intriguingly, although the results of this analysis might suggest that corticosteroids usage alone did not diminish the antitumor effectiveness of immunotherapy when focusing specifically on patients who received steroids for the management of irAE (*n* = 169), we found that the timing of steroid initiation in relation to the start of immunotherapy was a crucial indicator of disease progression.

While the median time for starting steroid treatment was 7.8 weeks, 23% of patients were introduced to steroids within the first 4 weeks of treatment. Curiously, those with early exposure (essentially, in the induction phase of immunotherapy treatment) had a significantly shorter median PFS (4.2 m) compared to those who were exposed in later stages (41.3 m) and even to those who were not exposed to steroids at all (8.7 m). The adjusted HR for progression or death was 3.2 for patients with early exposure compared with later exposure. This onset factor also produced a statistically significant result in multivariate analysis.

The observed significant association between early corticosteroid treatment and worsened PFS may suggest a potential causal relationship. While corticosteroids are unable to impede the robust antitumor immune response once this has been established through ICI administration, it is plausible that early exposure to corticosteroids during the brief induction phase of immunotherapy treatment (4 weeks) may interfere with the establishment of an effective immune response.

This analysis is likely exposed to the risk of an immortal time bias [55]. This risk (indicating that, during the period of observation, there is some interval during which the outcome event cannot occur and the patients are, therefore, “immortal”) pervades a remarkable proportion of oncological datasets aimed at a comparison; while in general, the degree of its relevance is still a matter of investigation [56], the present dataset does not seem to be strongly influenced by this bias.

The concept of early exposure to steroids during ICI treatment was also explored in preclinical studies utilizing mouse melanoma models. It was observed that the administration of immunotherapeutic inhibitors (ICIs) in combination with early steroidal treatment, but not late, resulted in the weakening of antitumor activity, promoting tumor regrowth and a reduction in CD8+ T cell proliferation. These results suggest that early steroidal exposure reduces memory CD8+ T cells, which play a critical role in the prolonged efficacy of ICIs [57].

An attempt to explore the correlation between the timing of steroidal treatment initiation and ICI efficacy has been conducted in other several retrospective works. Bai X et al. investigated the association between the use of high-dose steroids (≥60 mg prednisone equivalent per day) and survival in patients with advanced melanoma [58]. They concluded that early use, which they defined as within 8 weeks, was associated with poorer PFS/OS compared with later high-dose steroid use. Our study focused on very early usage within 4 weeks, as nearly a quarter of our patients (23%) were introduced to steroids within the first 4 weeks of treatment. Furthermore, we did not find any association between steroid dosage and PFS. Therefore, our findings are consistent with the results of this crucial study but reveal that even lower doses of steroids may have negative effects.

A study on 146 patients showed that early treatment with steroids (within the first month from the beginning of immunotherapy) had similar PFS curves to patients who were not exposed to steroids; however, patients exposed to steroids later in the course of treatment showed an interestingly longer PFS [43]. Another study on 151 patients with lung cancer found that 23% made early use of steroids, which was defined as the first 28 days after ICI initiation, and this early use was associated with poor disease control, PFS and OS [41,59]. A recently published work on 247 patients found that steroid exposure during the initial two months of immunotherapy adversely affected the Response Rate, PFS, and OS of metastatic patients [60].

While these findings, along with our results, imply a significant trend, a prospective clinical study is necessary to establish a more robust conclusion.

## 5. Conclusions

This study presents two key findings: (i) steroidal treatment during the immunotherapy priming phase (first 4 weeks) might have a deleterious effect on its efficacy; (ii) the immune activation associated with the development of irAEs can overcome the possible negative effect of steroids if given in later phases of the disease. To note, most irAEs generally occur within 10 weeks after ICI initiation, and a high fraction of patients are expected to be exposed to corticosteroids during ICI treatment, especially in dual therapy. This clinical reality highlights the importance of carefully weighing the use of steroid treatment for early onset irAEs, given its potential to negatively impact the effectiveness of immunotherapy.

## Figures and Tables

**Table 1:** Baseline demographic data and treatment disposition (*n* = 415).

Demographics	*n* (%)
Median age, years (range)	68 (12–99)
Male	241 (58%)
Baseline autoimmune disease ^1^	24 (5.8%)
Baseline immune-suppressive medications	5 (1.2%)
Histology	
Cutaneous	326 (78.5%)
Unknown	58 (14.1%)
Mucosal	20 (4.8%)
Acral	9 (2.1%)
Conjunctival	2 (0.5%)
AJCC 8th edition sub-stage	
M1a	118 (28.4%)
M1b	102 (24.6%)
M1c	144 (34.7%)
M1d	37 (8.9%)
Unknown	14 (3.4%)
Performance status	
ECOG 0–1	359 (89.7%)
ECOG 2–4	41 (10.2%)
BRAF status	
Wild type	259 (62.4%)
V600 mutant	122 (29.4%)
Unknown	37 (8.1%)
LDH level	
≤ UNL	181 (43.6%)
X1–2 UNL	63 (15.2%)
≥ X2 UNL	22 (5.3%)
Unknown	149 (35.9%)
Protocols	
Anti PD-1 monotherapy (Nivolumab or Pembrolizumab)	271 (65.3%)
ipilimumab + nivolumab	101 (24.3%)
Anti CTLA-4 monotherapy	43 (10.4%)
Treatment status	
Ongoing	44 (10.6%)
Stopped d/t progression	193 (46.5%)
Stopped d/t toxicity	86 (20.7%)
Stopped d/t major response	50 (12.1%)
Stopped d/t other reasons	42 (10.1%)
Response-Rate	
Ipilimumab + nivolumab	57 (59.4%)
Anti PD-1 monotherapy	148 (58.3%)
Anti CTLA-4 monotherapy	8 (20.5%)
Median PFS	
Ipilimumab + nivolumab	12.8 m
Anti PD-1 monotherapy	12.5 m
Anti CTLA-4 monotherapy	3.4 m

Abbreviations: AJCC-American Joint Committee on Cancer, ECOG-Eastern Cooperative Oncology Group, LDH—Lactate dehydrogenase, UNL—Upper Normal Limit, PD-1—Programmed Death-1, CTLA-4—cytotoxic T-lymphocyte-associated protein 4, PFS- Progression Free Survival. ^1^ Inflammatory bowel disease—4, Gout—4, immune thrombocytopenia—2, psoriasis—2, ankylosing spondylitis—2, atopic dermatitis—1, bell’s palsy—1, bullous pemphigoid—1, dermatomyoisitis—1, hashimoto thyroiditis—1, rheumatoid arthritis—1, sjogren’s syndrome—1, vitiligo—1.

**Table 2:** Univariable and Multivariable analysis for Progression-Free survival.

Univariable Analysis			
	Hazard Ratio for PFS	95% CI	*p*-Value
Age	1.01	0.99–1.01	0.7
Sex	1.01	0.79–1.28	0.9
Cutaneous histology	0.69	0.48–0.99	0.045
M sub-stage	1.19	1.05–1.34	0.004
ECOG performance status	1.29	1.11–1.49	0.001
BRAF V600 mutation	0.88	0.67–1.16	0.4
Relative LDH level	1.24	1.16–1.34	<0.001
anti PD-1 containing treatments vs. anti CTLA-4 alone	0.75	0.60–0.94	0.014
irAEs	0.46	0.35–0.61	<0.001
Dose of steroids	0.98	0.93–1.02	0.3
“early” vs. “late” onset of steroids *	3.29	1.99–5.43	<0.001
**Multivariable Analysis**			
	**Hazard Ratio for PFS**	**95% CI**	***p*** **-Value**
Cutaneous histology	0.74	0.42–1.32	0.3
M sub-stage	1.28	1.04–1.57	0.02
ECOG performance status	1.04	0.82–1.31	0.7
Relative LDH level	1.19	1.08–1.32	0.001
anti PD-1 containing treatments vs. anti CTLA-4 alone	0.68	0.46–0.99	0.049
irAEs	0.48	0.31–0.75	0.001
“early” vs. “late” onset of steroids *	2.45	1.26–4.74	0.008

Abbreviations: PFS—Progression Free Survival, CI—Confidence-Interval, M sub-stage- Metastasis sub-stage, ECOG—Eastern Cooperative Oncology Group, LDH—Lactate dehydrogenase, PD-1—Programmed Death-1, CTLA-4- Cytotoxic T Lymphocyte Antigen-4, irAE- immune related adverse events, “Late” onset of steroids—onset within more than 4 weeks from immunotherapy initiation, “Early” onset of steroids—onset within the first 4 weeks of immunotherapy. * not adjusted with landmark analysis.

## Data Availability

The data presented in this study are available on request from the corresponding author.
